# Serum brain-derived neurotrophic factor levels in patients with schizophrenia and methamphetamine addiction: correlation with Mini-Mental State Examination (MMSE)

**DOI:** 10.25122/jml-2022-0250

**Published:** 2023-05

**Authors:** Saja Mahir Mohammed, Zainab Hassan Hashim, Mahir Mohammed Hussein, Qasim Sharhan Al-Mayah

**Affiliations:** 1. Department of Pharmacy, Osol Aldeen University College, Baghdad, Iraq; 2. Department of Physiology, College of Medicine, Al-Nahrain University, Baghdad, Iraq; 3. Ibn-Rushed Psychiatric Teaching Hospital, Baghdad, Iraq; 4. Medical Research Unit, College of Medicine, Al-Nahrain University, Baghdad, Iraq

**Keywords:** brain-derived neurotrophic factor (BDNF), schizophrenia, methamphetamine addiction

## Abstract

Methamphetamine use can induce psychosis resembling acute schizophrenia spectrum psychosis, making it challenging to differentiate between the two based on symptoms alone. Brain-derived neurotrophic factor (BDNF) exerts a critical role in hippocampal neural plasticity, influencing critical cognitive functions such as memory and learning. This study aimed to determine the role of serum BDNF levels in schizophrenia and methamphetamine addiction. A case-control study was conducted involving 50 patients with schizophrenia, 50 patients with methamphetamine addiction, and 50 healthy control subjects recruited from Ibn-Rushed Psychiatric Teaching Hospital in Baghdad. Cognitive impairment was assessed using the Mini-Mental State Examination (MMSE), while serum BDNF levels were measured using ELISA following standardized protocols. The findings revealed significantly lower median levels of BDNF (0.36 pg/ml) in patients with schizophrenia compared to both the control group (0.51 pg/ml) and the methamphetamine group (0.72 pg/ml). Moreover, there was a significant difference observed between the methamphetamine group and the control group. At a cut-off value of BDNF=0.37 pg/ml, the sensitivity and specificity of BDNF in differentiating between schizophrenia and methamphetamine addiction were 84% and 70%, respectively. Serum level of BDNF could be used to differentiate between schizophrenia and methamphetamine addiction when clinical distinctions are challenging to detect.

## INTRODUCTION

Schizophrenia remains a significant global concern, with behavioral and mental illnesses accounting for a substantial disability-adjusted life year (DALY) burden worldwide. Among these conditions, schizophrenia alone contributes to approximately 0.6% of the total DALY burden [1]. Over the past three decades, the neural development theory has emerged as a prominent framework for understanding the etiology of schizophrenia, as proposed by Weinberger, Murray, and Robin [2]. The use of methamphetamine is widespread in the total population and clients with psychiatric diseases. Methamphetamine use has been associated with the emergence of psychotic symptoms that closely resemble those observed in acute schizophrenia spectrum psychosis. There was a conflict for utilizing methamphetamine-induced psychotic disease as a model for primary psychosis diseases. The discrimination between these two types of psychotic disorders solely based on acute signs and symptoms poses significant challenges. Nonetheless, acute psychosis triggered by methamphetamines tends to have a faster recovery and more comprehensive resolution than schizophrenia. Schizophrenic spectrum disorder and methamphetamine-induced psychotic disorder are interconnected by the identification of multiple susceptibility genes that are implicated in both conditions [3].

Brain-derived neurotrophic factor (BDNF), which plays a crucial role in neuronal differentiation, growth, and synaptic plasticity within the hippocampus, has been implicated in various cognitive functions such as memory and learning [4,5]. BDNF is also dysregulated in several psychiatric disorders, including bipolar disorder, schizophrenia, depression, and autism spectrum disorder [6,7]. Furthermore, BDNF levels are elevated in recovering methamphetamine addicts, suggesting its potential as a biomarker. Recent studies have explored the correlation between serum BDNF levels and MMSE, revealing a significant positive association with Alzheimer's disease [8]. Similar findings have been reported in patients with schizophrenia by Binford et al. [9]. The present study aimed to investigate the role of BDNF levels in individuals with schizophrenia and methamphetamine addiction, as well as their correlation with MMSE scores.

## Material and Methods

### Study groups

This case-control study recruited 50 patients with schizophrenia and 50 participants with methamphetamine (crystal) addiction from Ibn-Rushed Psychiatric Teaching Hospital in Baghdad. The diagnosis of schizophrenia was made by specialist psychiatrists based on the criteria outlined in the Diagnostic and Statistical Manual of Mental Disorders (DSM-5), along with cognitive and memory tests approved by the hospital. The diagnosis of methamphetamine addiction was determined by specialist psychiatrists using clinical data and the criteria for addiction in the International Statistical Classification of Diseases and Related Health Problems 10th Revision (ICD-10) [4]. Additionally, 50 age- and sex-matched healthy participants were randomly selected as the control group from private clinics. All participants underwent a thorough physical examination and various investigations, including assessment of age, gender, body mass index (BMI), lipid profile, and complete blood count.

### Sample collection and BDNF measurement

All participants were instructed to fast for 8 hours before blood sampling. The following morning, approximately 5 mL of venous blood was collected from each patient, with 2 mL reserved in an ethylenediaminetetraacetic acid (EDTA) tube and the remaining 3 mL in a gel tube. The serum was separated and stored in Eppendorf tubes at −20°C until further use. Sandwich enzyme-linked immunosorbent assay (ELISA) (Sunlong, China) was used to measure the serum level of BDNF following the manufacturer’s protocols.

### Biochemical tests

In addition to BDNF, fasting blood sugar levels and lipid profiles, including triglycerides (TG), total cholesterol (TC), high-density lipoprotein (HDL), and low-density lipoprotein (LDL), were measured according to the standard protocols.

### Mini-Mental State Examination (MMSE)

Cognitive impairment was assessed in all patients using the MMSE to correlate the score with BDNF levels, which play a role in learning, memory, and cognition. The Arabic version of the MMSE was employed, and it was included in the diagnostic interview schedule to determine the cognitive dysfunction in all participants. The MMSE is an 11-item questionnaire that measures five areas of cognitive function, including calculation, registration, orientation, attention, recall, and language. The maximum score is 30, and scores below 25 indicate impairment. A cut-off point of 23 distinguishes patients with definite cognitive impairment from healthy subjects in a reliable manner [4]. The MMSE test can be completed in 5-10 minutes and is routinely used.

### Statistical analysis

Data analysis and management were performed using SPSS version 25 software, and Microsoft Excel was used for graphical presentations. Descriptive statistics, including mean scores, median and range, frequency and percent, were used for data analysis. ANOVA (F test) and Kruskal Wallis tests were employed to assess differences in mean scores across categorical variables. The Chi-square test was used to analyze qualitative data. Receiver operating characteristic curve (ROC) analysis was used to determine the discriminative value of BDNF between schizophrenia and methamphetamine addiction. Pearson's correlation test was used to explore the correlation between BDNF, MMSE scores, and other parameters. A significance level of 0.05 or less was considered statistically significant.

## Results

### Differences in age and gender

The mean age was significantly different among the study groups (p<0.001). The mean age of the methamphetamine group was significantly lower than that of both the control group and the schizophrenia group (Table 1). However, there was no significant difference in mean age between the control group and the schizophrenia group. Regarding gender, the control group consisted of 36 (72.0%) males and 14 (28.0%) females, the schizophrenia group included 24 (48%) males and 26 (52%) females, and the methamphetamine group included 44 (88.0%) males and 6 (12.0%) females. The proportions of males and females varied significantly among the study groups (p<0.001).

**Table 1.** Demographic characteristics of participants

Characteristics	Control n = (50)	Schizophrenia n = (50)	Methamphetamine n = (50)	p-value
**Age (years)**				
Mean ± SD	37.34±8.97^a^	37.24±12.48^a^	27.36±6.71^b^	< 0.001 A^**^
Range	22-57	19-62	20-50	
**Gender**				
Male, n (%)	36 (72.0 %)	24 (48.0 %)	44 (88.0 %)	< 0.001 B^**^
Female, n (%)	14 (28.0 %)	26 (52.0 %)	6 (12.0 %)	
**MMSE**				
Mean ± SD	25.60±1.50^a^	19.12±1.90^b^	18.84±1.75^b^	< 0.001 A^**^
Range	24-29	16-23	16-22	

n: frequency of cases; SD: Standard Deviation, A: ANOVA, B: Chi-square test ^**^Highly significant at p ≤ 0.01; a, b: different small letters indicate levels of significance after post hoc test

### Mini-Mental State Examination (MMSE)

The mean MMSE score was significantly different among the study groups (p<0.001). The control group had a mean MMSE score of 25.60±1.50, significantly higher than that of the schizophrenia group (19.12±1.90) and the methamphetamine group (18.84±1.75). However, there was no significant difference between the schizophrenia group and the methamphetamine group.

### Mini-Mental State Examination

**Figure 1.**
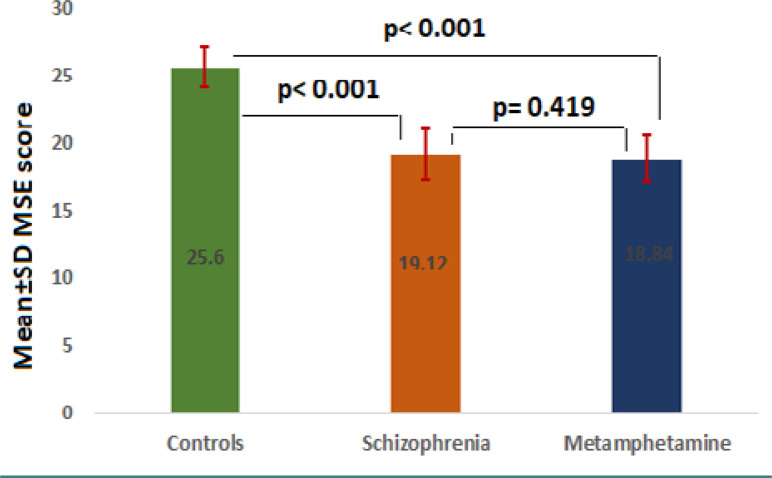
Comparison of MMSE among control, schizophrenia, and methamphetamine groups (bar chart)

Figure 1 illustrates the comparison of Mini–Mental State Examination (MMSE) scores among three groups: the control group, the schizophrenia group, and the methamphetamine addiction group. The mean MMSE score in the control group was 25.60±1.50, significantly higher than in the schizophrenia group (19.12±1.90) and the methamphetamine addiction group (18.84±1.75). However, there was no significant difference observed between the two patient groups.

### Biochemical characteristics

Patients in the schizophrenia group had higher fasting blood sugar (FBS) levels compared to the control group, and the control group had higher FBS levels than the methamphetamine group, with highly significant differences (p<0.001). The control group displayed higher levels of high-density lipoprotein (HDL) compared to the schizophrenia and methamphetamine groups, with highly significant differences (p<0.001). However, patients in the schizophrenia and methamphetamine groups demonstrated higher levels of low-density lipoprotein (LDL) compared to the control group, with highly significant differences (p<0.001). There were no significant differences in cholesterol and triglyceride levels among the study groups (Table 2).

**Table 2.** Biochemical characteristics of participants

Characteristics	Control n = (50)	Schizophrenia n = (50)	Methamphetamine n = (50)	p-value
FPG (mg/dl)				
Mean ± SD	98.24±8.65^a^	105.20±9.45^b^	89.68±5.75	< 0.001 O**
Range	90-120	94-126	75-99
Cholesterol (mg/dl)				
Mean ± SD	212.13±52.77	194.27±34.98	203.68±53.66	< 0.001 B**
Range	146-370	137-286.9	137-370	NS
Triglycerides (mg/dl)				
Mean ± SD	164.45±51.81	159.63±35.83	148.67±47.81	0.212 O
Range	112-344	101-244	70-284	NS
HDL (mg/dl)				
Mean ± SD	44.06±12.71a	27.46±6.63b	28.61±6.03b	< 0.001 O**
Range	23-67	18-44	19-41.6
LDL (mg/dl)				
Mean ± SD	123.56±11.61^a^	152.23±34.20^b^	157.43±49.34^b^	< 0.001 O**
Range	102-160	100.7-224.2	90.8-280.2

n: frequency of cases; SD: Standard Deviation, A: ANOVA, B: Chi-square test ^**^Highly significant at p ≤ 0.01; NS: not significant; a, b: Small different letters indicate the level of significance after the post hoc test

### Brain-derived neurotrophic factor

**Figure 2.**
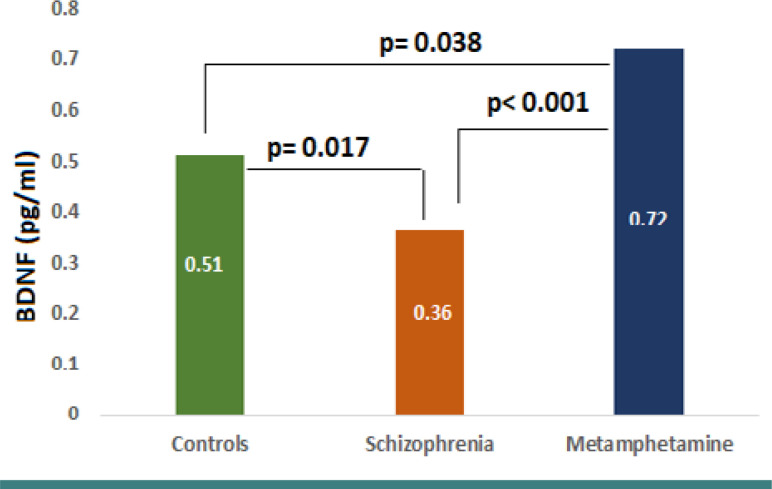
Differences in BDNF levels among control, schizophrenia, and methamphetamine groups

The serum levels of BDNF were not normally distributed. Participants with schizophrenia had a significantly lower median level of BDNF (0.36 pg/ml) compared to the control group (0.51 pg/ml) and the methamphetamine group (0.72 pg/ml). There was also a significant difference between the methamphetamine and control groups (Figure 2).

### Diagnostic value of brain-derived neurotrophic factor

**Figure 3.**
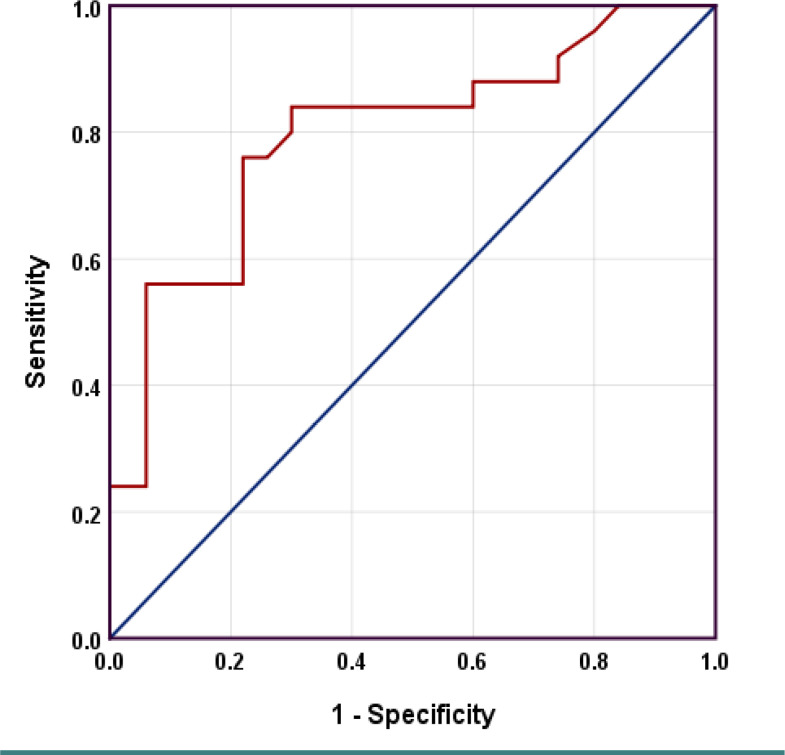
Receiver operating curve for the diagnostic power of BDNF differentiation between subjects with schizophrenia and methamphetamine addiction

The receiver operating characteristic (ROC) curve analysis indicated that BDNF had a diagnostic power in differentiating between patients with schizophrenia and methamphetamine addiction. The area under the curve (AUC) was 0.796 (95% CI=0.708-0.885, p<0.001). At a cut-off point of BDNF=0.37 pg/ml, the sensitivity and specificity of the test were 84% and 70%, respectively (Figure 3).

### Correlation of BDNF and MMSE with other variables

**Figure 4.**
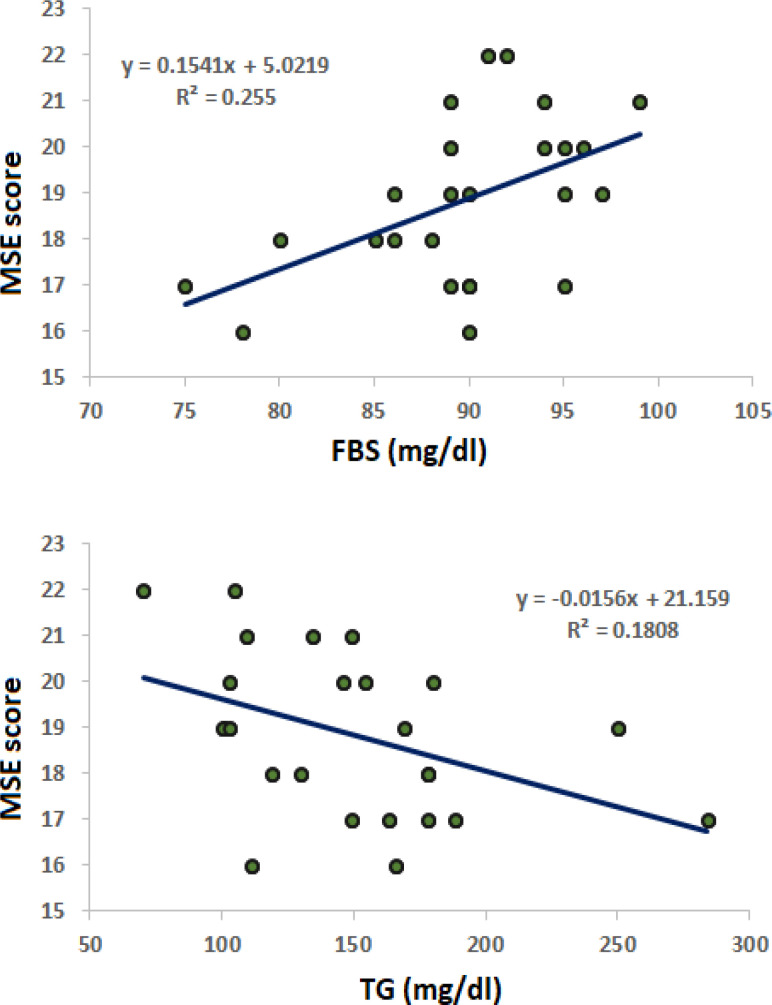
Scatter plot and regression line between FBS and TG with MMSE in patients with methamphetamine addiction

In the schizophrenia group, MMSE and BDNF had no significant correlation with any of the included variables in this group. However, in the methamphetamine addiction group, MMSE showed a significant positive correlation with FBS (r=0.505, p<0.001) and a significant negative correlation with TG (r=-0.425, p=0.002), as explained in Table 3 and Figure 4.

**Table 3.** Pearson's correlation coefficient (r) for MMSE and BDNF with other variables in patients with schizophrenia and methamphetamine addiction

Variable	Schizophrenia				Amphetamine addiction			
MMSE		BDNF		MMSE		BDNF	
r	p-value	r	p-value	r	p-value	r	p-value
Age	-0.257	0.071	0.095	0.513	-0.113	0.435	-0.187	0.193
FBS	-0.067	0.643	-0.180	0.210	0.505	<0.001	0.219	0.126
TC	0.106	0.464	0.039	0.789	-0.013	0.927	0.134	0.155
TG	0.083	0.567	0.009	0.950	-0.425	0.002	0.133	0.358
HDL	-0.095	0.513	-0.218	0.128	0.118	0.422	0.076	0.588
LDL	-0.025	0.885	-0.077	0.595	-0.116	0.421	-0.063	0.666
BNDF	-0.060	0.679			0.080	0.581		

## Discussion

This study investigated the role of serum brain-derived neurotrophic factor (BDNF) levels in individuals with schizophrenia and methamphetamine addiction and their correlation with Mini-Mental State Examination (MMSE) scores. The findings revealed significantly lower BDNF levels in patients with schizophrenia compared to both the control group and the methamphetamine group.

According to Shakeri et al. [10], the mean score of mental status was significantly lower in patients with methamphetamine addiction in comparison with the control group. Similarly, Scott et al. [11] found that methamphetamine users (compared to non-substance users) had deficiencies in learning, executive function, memory, processing speed, and language to a lesser extent. In addition, Kalechstein et al. [12] found that methamphetamine addicts demonstrated significant impairments in memory, attention, verbal learning, and psychomotor speed compared to non-users. Jovanovski et al. [13] investigated the neurological impairments in users of cocaine. The study included 481 cocaine users and 586 non-users as participants. The findings revealed that cocaine usage had the greatest effect, with those using cocaine suffering from sensory motor skills, visual memory, attention deficit disorder, language functions, and operational memory. In addition, Rogers and Robbins [14] conducted a study of neuropsychological abnormalities related to chronic substance use and concluded that substance use is linked to attention and memory impairment. According to the findings of the present study, disruption to specific brain areas might induce attention deficit disorder, with the front-striatal connections being one of the most vulnerable areas [15]. When individuals with substance use disorders are required to focus on tasks that demand alertness, they encounter a range of challenges [16]. Studies have shown decreased activity in the anterior-visual and anterior-frontal regions of the brain in individuals with addiction compared to non-users [17, 18]. According to Simon et al. [18], the loss of dopaminergic neurons in front striatal areas, including the striatum and front cingulate cortex, is linked to decreased cognitive control and selective attention in methamphetamine addicts. Furthermore, an animal study found that methamphetamine use causes hippocampal inhibitory neurons, leading to abnormalities in hippocampus-dependent cognitive activities [19]. Drugs such as opium can impact the neurotransmitters involved in the release of dopamine in the striatum, as well as the release of norepinephrine, serotonin, and glutamic acid. These alterations in neurotransmitter function can result in psychological and cognitive difficulties, as well as functional changes in the brain [20]. Furthermore, cognitive issues in individuals who use substances could be caused by the substance’s direct impacts on the brain, in addition to the impacts on the frontal-hippocampal fragments and cerebral systems [21].

Our study revealed significant variations in mean fasting blood sugar (FBS) levels among the study groups. The schizophrenia group showed the highest FBS concentration, while the methamphetamine group exhibited the lowest levels. These findings suggest a potential association between altered glucose metabolism and schizophrenia, as well as a possible link between excessive glucose consumption and methamphetamine addiction. However, further research is necessary to investigate the underlying mechanisms and establish causal relationships. The prevalence of type 2 diabetes (T2DM) is two to five times higher in individuals with schizophrenia compared to the general population [21]. A sedentary lifestyle, a poor diet, and obesity are common risk factors for T2DM in patients with schizophrenia, particularly in the early stages of the illness. Individuals with schizophrenia frequently have a low social and monthly income level, limiting their ability to make appropriate lifestyle decisions [22]. Antipsychotic medicines raise the incidence of T2DM directly and indirectly by altering insulin sensitivity and promoting weight gain. Treatment for patients with schizophrenia should include lifestyle change treatments for diabetes prevention [22]. Concerning lipid profiles in the present study, there was no significant variation in mean serum cholesterol and triglyceride levels. However, serum HDL had a significantly lower mean, and serum LDL had a significantly higher mean in patients with schizophrenia and methamphetamine addiction than in the control group, suggesting that some form of dyslipidemia may be linked to schizophrenia and methamphetamine addiction. Several previous authors reported increased dyslipidemia associated with schizophrenia [23, 24]. The authors have linked dyslipidemia to the adverse effects of pharmacological agents used to control symptoms of schizophrenia. With respect to methamphetamine, previous experimental work has shown that prolonged methamphetamine therapy can increase the formation of atherosclerotic plaque [25, 26].

In our study, we observed a significant decrease in brain-derived Neurotrophic factor (BDNF) levels in the schizophrenia group, while the methamphetamine addiction group showed higher BDNF levels compared to the healthy control group. It is important to note that research on peripheral BDNF levels in individuals with schizophrenia has yielded varied results. While most studies have reported decreased peripheral BDNF levels, some articles have reported elevated BDNF levels in individuals with schizophrenia. These discrepancies in findings could be attributed to factors such as the characteristics of the studied populations (e.g., treatment-naïve individuals, medicated vs. unmedicated individuals) or differences in sampling sources (e.g., serum vs. serum protein) [27-30]. Although this variation in peripheral BDNF levels is an interesting outcome, it is still unclear whether peripheral levels accurately reflect BDNF levels in the central nervous system. It is possible that measuring BDNF levels in the brain itself may provide a better understanding of the role of BDNF in schizophrenia. Consequently, peripheral BDNF levels as a reliable biomarker for schizophrenia are currently not well-established [27].

In one meta-analysis of 16 studies, Green et al. [28] found solid evidence of reduced blood levels of BDNF in patients with schizophrenia, whether newly diagnosed or on treatment. This reduction in the BDNF level may present a diagnostic biomarker in addition to its role in evaluating the disease prognosis. Kim et al. [31] found that methamphetamine addiction was associated with significantly higher serum BDNF levels compared to healthy subjects, indicating a potential role of BDNF in the neural toxicity of methamphetamine. Similarly, Ren et al. [32] reported that methamphetamine addicts had higher baseline serum BDNF levels than controls. However, after one month of withdrawal, the BDNF levels in methamphetamine addicts decreased and became similar to the control group. This suggests that high BDNF concentrations may be linked to withdrawal and addiction, potentially protecting against the harmful effects of methamphetamine.

Our study found a significant positive correlation between MMSE scores and fasting blood sugar (FBS) and triglyceride levels in the methamphetamine addiction group. It is well-known that diabetes mellitus is associated with accelerated atherosclerosis [33]. A recent large-scale population-based study conducted in China demonstrated that MMSE scores decline in elderly individuals with type 2 diabetes mellitus (T2DM), suggesting that metabolic changes associated with diabetes may impact cognitive abilities in patients with chronicity of the disease [34].

## Conclusion

In conclusion, the serum level of BDNF can be a useful marker for distinguishing between schizophrenia and methamphetamine addiction, particularly in cases where clinical differences are challenging to detect. No direct correlation was found between BDNF and MMSE in both individuals with schizophrenia and those with methamphetamine addiction.
